# Traditional Chinese medicine ginseng regulates neurotrophic factors to improve neurodegenerative diseases: A potential strategy for Alzheimer's disease treatment

**DOI:** 10.1016/j.jgr.2026.101078

**Published:** 2026-06-19

**Authors:** Qucheng Huang, Yining Xie, Xiaojun Zhang, YuanYuan Zuo, Leiyi Wang, Hewei Xu, Miao Yu, Chang Liu

**Affiliations:** a College of Pharmacy, Beihua University, Jilin, Jilin, 132013, PR China; b Department of Physical and Chemical Inspection, Nanguan District Center for Disease Control and Prevention, Changchun, jilin, 130022, PR China; c State key Laboratory of Electroanalytical Chemistry, Changchun Institute of Applied Chemistry, Changchun, jilin, 130022, PR China

**Keywords:** Ginseng, Alzheimer's disease, Neurotrophic factors, BDNF/TrkB signaling pathway, Traditional medicine

## Abstract

Alzheimer's disease (AD) is a neurodegenerative disorder characterized by memory impairment and progressive cognitive decline. Its core pathological mechanisms include β-amyloid deposition, abnormal tau phosphorylation, neuroinflammation, and decreased levels of neurotrophic factors. In recent years, the deficiency of neurotrophic factors has been recognized as a key driver of impaired neuronal survival and synaptic dysfunction. Consequently, restoring or enhancing neurotrophic factor signaling has emerged as a major therapeutic target for AD. As a traditional medicinal plant, ginseng exhibits multi-targeted, multi-level neuroprotective effects. Its active components—including ginsenosides, ginseng polysaccharides, and ginseng proteins—have been demonstrated to promote neuronal survival, inhibit apoptosis, and enhance synaptic plasticity by upregulating signaling pathways such as BDNF/TrkB and NGF/TrkA. Additionally, ginseng suppresses inflammatory responses and oxidative stress, thereby indirectly correcting neurotrophic factor imbalances and demonstrating significant neuroprotective potential. This review summarizes recent advances in ginseng's regulation of neurotrophic factor levels and signaling pathways, focusing on its molecular mechanisms for improving neuronal dysfunction and delaying AD progression. It also outlines future research strategies and prospects for clinical translation. In summary, ginseng's neuroprotective effects achieved through regulating the neurotrophic factor network offer a potential natural drug intervention strategy for AD treatment.

## Highlights

• Constructed a neurotrophic factor network map revealing ginseng’s multi-target synergistic effects in AD.
• Proposed the “ginseng–gut–brain axis” with single-cell and nanodelivery-based research strategies.
• Integrating traditional medicine, neuropharmacology, nanotechnology and multi-omics to form an interdisciplinary research strategy.
• Established a translational pathway from mechanisms to formulation and clinical application.

## Graphical abstract

**Figure:**
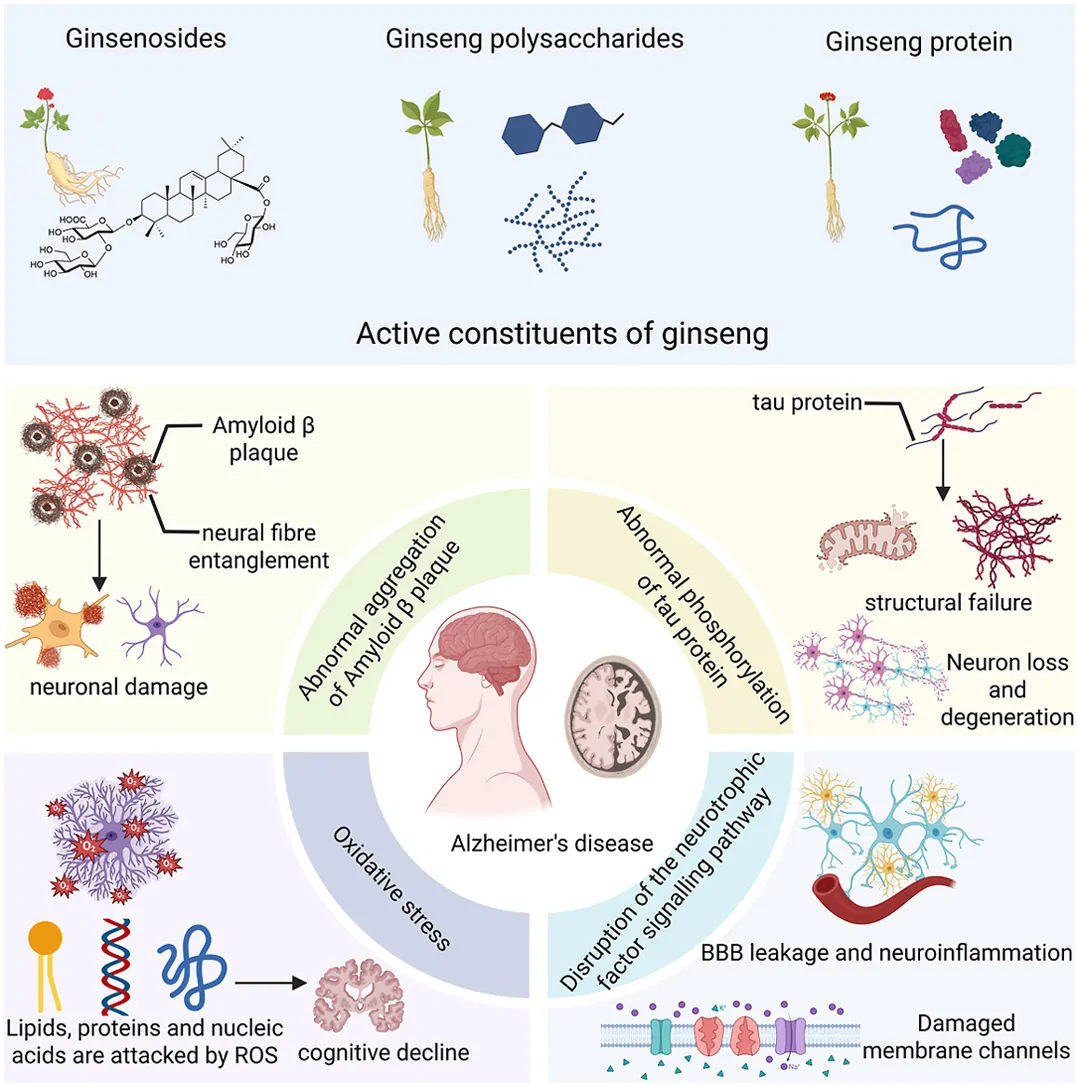

## Abbreviations

AAV: adeno-associated virus
Aβ: amyloid beta
ABAD: alcohol dehydrogenase bound to Aβ on mitochondria
AchE: acetylcholine
AD: Alzheimer's disease
APP: amyloid precursor protein
ApoE2: Apolipoprotein E2
ApoE4: Apolipoprotein E4
ARE: antioxidant response element
ATP: adenosine triphosphate
BCL2: B-cell Lymphoma 2
BACE1: β-site APP cleaving enzyme 1
BAX: Bcl-2 associated X protein
BFCNs: basal forebrain cholinergic neurons
BDNF: brain-derived neurotrophic factor
CAT: catalase
ChAT: choline acetyltransferase
CK: compound K
CNTF: ciliary neurotrophic factor
CREB: cAMP-response element binding protein
Drp1: dynamin-related protein 1
ELAVL2: ELAV like RNA binding protein 2
ERK: extracellular regulated protein kinases
GABA: gamma-aminobutyric acid
GPx: glutathione peroxidase
HNE: 4-hydroxy-2-nonenal
HO-1: oxygenase-1
IL-6: interleukin-6
IL-10: interleukin-10
LTP: long-term potentiation
MANF: mesencephalic astrocyte-derived neurotrophic
MAPK: Mitogen-activated protein kinase
MDA: malondialdehyde
MyD88: myeloid differentiation primary response 88
NF-κB: nuclear factor kappa-B
NGF: nerve growth factor
Nrf2/ARE: nuclear factor E2-related factor 2/antioxidant response element
NT-3: neurotrophic factor-3
NT-4/5: Neurotrophin-4/5
NTFs: neurotrophic factors
OA: oleanolic acid-type
p75NTR: nerve growth factor receptor p75
PI3K/Akt: phosphoinositide 3-kinase/protein kinase B
PKC: protein kinase C
PLCγ: phospholipase C gamma
PPARγ: peroxisome proliferator-activated receptor gamma
PPD: protopanaxadiol-type
PPT: protopanaxatriol-type
ROS: reactive oxygen species
RNS: reactive nitrogen species
RRM3: third RNA recognition motif
SIRT1/PGC-1α: Sirtuin 1/peroxisome proliferator-activated receptor gamma coactivator 1 alpha
S1P: sphingosine 1-phosphate
SOD: superoxide dismutase
TLR4: toll like receptors 4
TNF-α: tumor necrosis factor-alpha

## 1 Introduction

Alzheimer's disease (AD) is a neurodegenerative disorder clinically defined by memory impairment, cognitive decline, and behavioral changes. Pathologically, the ATN framework characterizes AD by β-amyloid (Aβ) deposits and phosphorylated tau tangles, along with neurodegeneration, leading to progressive dysfunction and reduced quality of life and social functioning [1]. Due to global population ageing, AD incidence rises yearly; over 50 million people currently live with AD, two-thirds in low- and middle-income countries—a proportion expected to grow. By 2050, cases will reach nearly 139 million [2]. AD's high incidence and chronic disability burden families, public health systems, and socioeconomics, making effective interventions a critical global priority [3].

The etiology and pathogenesis of AD are highly complex and have not yet been fully elucidated; however, extensive research indicates that several core pathological processes play a critical role in disease progression. First, Aβ is generated through the cleavage of APP by β- and γ-secretases and forms toxic oligomers and plaques through abnormal aggregation. This is considered one of the pathological hallmarks of AD. When there is an imbalance between Aβ production and clearance, abnormal aggregation occurs, triggering various toxic effects [4]. Second, patients with AD exhibit abnormal hyperphosphorylation of tau protein, meaning that excessive phosphate groups are attached to the tau protein molecules, thereby altering their three-dimensional structure. Hyperphosphorylated tau protein is unable to bind effectively to microtubules, leading to microtubule instability and even disassembly. As a result, synaptic function is impaired and neuronal degeneration is triggered, thereby exacerbating the condition in AD patients [5]. Oxidative stress is also a key pathological mechanism in AD. By attacking various macromolecules within cells, oxidative stress directly drives the progression of AD. Attacking lipids: It generates 4-hydroxy-2-nonenal (HNE), malondialdehyde (MDA), and F2-isoprostaglandin. HNE disrupts ion channels and transporters, leading to calcium homeostasis imbalance and inducing neuronal apoptosis. Protein damage: **Reactive oxygen species (ROS)** induce protein carbonylation, nitrosylation, and cross-linking, leading to loss of protein function and misfolding. The aforementioned Aβ toxicity, abnormal tau phosphorylation, and oxidative stress all directly impair the expression and signaling of neurotrophic factors, causing the collapse of the nutritional supportsystem essential for neuronal survival and plasticity, thereby accelerating the progression of AD. The aforementioned Aβ deposition, tau hyperphosphorylation, and oxidative stress all directly impair the expression and signaling of neurotrophic factors, leading to neurotrophic dysfunction. This dysfunction not only weakens the nutritional supportessential for neuronal survival and the maintenance of synaptic plasticity but also further amplifies the damaging effects of Aβ toxicity, tau pathology, and oxidative stress. Consequently, it accelerates neuroinflammation, synaptic loss, and neuronal death, serving as a core driver of AD progression. Therefore, modulating neurotrophic factors signaling pathways has become a key strategy for AD intervention [6,7].Attacking nucleic acids causes DNA breaks and impairs oxidative phosphorylation, reducing ATP production and increasing **ROS** leakage, thus creating a vicious cycle [8]. Mitochondrial dysfunction and structural abnormalities in AD disrupt the electron transport chain, leading to severe ATP deficits. This energy deprivation in neurons impairs electrical activity, synaptic transmission, and repair mechanisms, directly causing cognitive decline and ultimately neuronal death [9]. Additionally, evidence shows that reduced brain-derived neurotrophic factor (BDNF) and impaired signaling are key drivers of AD neurodegeneration. In AD patients, BDNF mRNA and protein are significantly decreased in the hippocampus, temporal, parietal, and olfactory cortices. BDNF activates downstream pathways (PI3K/Akt, ERK/CREB, PLCγ/PKC) via its receptor TrkB, supporting neuronal survival, synaptic plasticity, and memory. BDNF also suppresses NF-κB-mediated inflammation; its absence exacerbates neuroinflammation, further damaging neurons [6]. Against the pathological backdrop of AD, dysfunction of the nerve growth factor (NGF) signaling pathway is particularly prominent. NGF primarily promotes the survival and axonal growth of cholinergic neurons in the basal forebrain through the TrkA receptor; however, this region undergoes significant degeneration and synaptic loss as early as the initial stages of AD. Concurrently, abnormal interactions between NGF and the p75 receptor can exacerbate Aβ-induced neuronal apoptosis. Therefore, modulating the NGF/TrkA signaling axis is critical for rescuing AD-related cholinergic deficits, inhibiting tau protein hyperphosphorylation, and preserving synaptic plasticity. Ciliary neurotrophic factor (CNTF) activates the JAK/STAT signaling pathway *via*the CNTFRα/LIFR/gp130 receptor complex; Downregulation of this pathway in AD is closely associated with impaired adult hippocampal neurogenesis and cognitive decline. Enhanced CNTF signaling not only promotes the differentiation of neural stem cells into functional neurons to counteract neural stem cell depletion caused by Aβ deposition but also exerts anti-neuroinflammatory effects by inhibiting excessive microglial activation and reducing the release of pro-inflammatory cytokines [10,11]. Clinical and basic research consistently show reduced levels of these factors in AD patients, and their signaling dysfunction strongly correlates with cognitive impairment [7]. Thus, restoring neurotrophic factor levels or enhancing their signaling has become a major focus in recent AD research.

Ginseng offers unique advantages in AD interventions. Modern research shows that ginseng and its primary active components have significant neuroprotective effects. Ginsenosides Rg1, Rb1, and Rd improve learning and memory impairments [12]. Ginseng polysaccharides potently inhibit the pro-inflammatory MAPK/NF-κB pathway, activate the p62-Nrf2-Keap1 antioxidant pathway, and induce autophagy. Ginseng protein exhibits anti-apoptotic and immunomodulatory functions, providing multifaceted neuroprotective support [13].

**Notably, the mechanism** underlying the interaction between human cells and neurotrophic factors has increasingly become a research focus in recent years. Numerous experimental studies have shown that ginsenoside RK3 promotes neurogenesis in AD by activating the PI3K/Akt signaling pathway, thereby improving cognitive deficits [14]. Ginsenoside Rg1 enhances the activity of antioxidant enzymes by activating the Nrf2/HO-1 pathway, thereby reducing levels of ROS and MDA, while simultaneously improving cellular antioxidant capacity [15]. Some studies have also shown that ginseng extract can simultaneously influence the expression of BDNF and NGF, exerting a synergistic effect by modulating the PI3K/Akt, ERK/CREB, and Nrf2/HO-1 pathways. This ability to improve AD pathology by regulating the neurotrophic factor network provides a solid mechanistic basis for the use of ginseng in the prevention and treatment of AD [13,15,16].

**Fig. 1:**
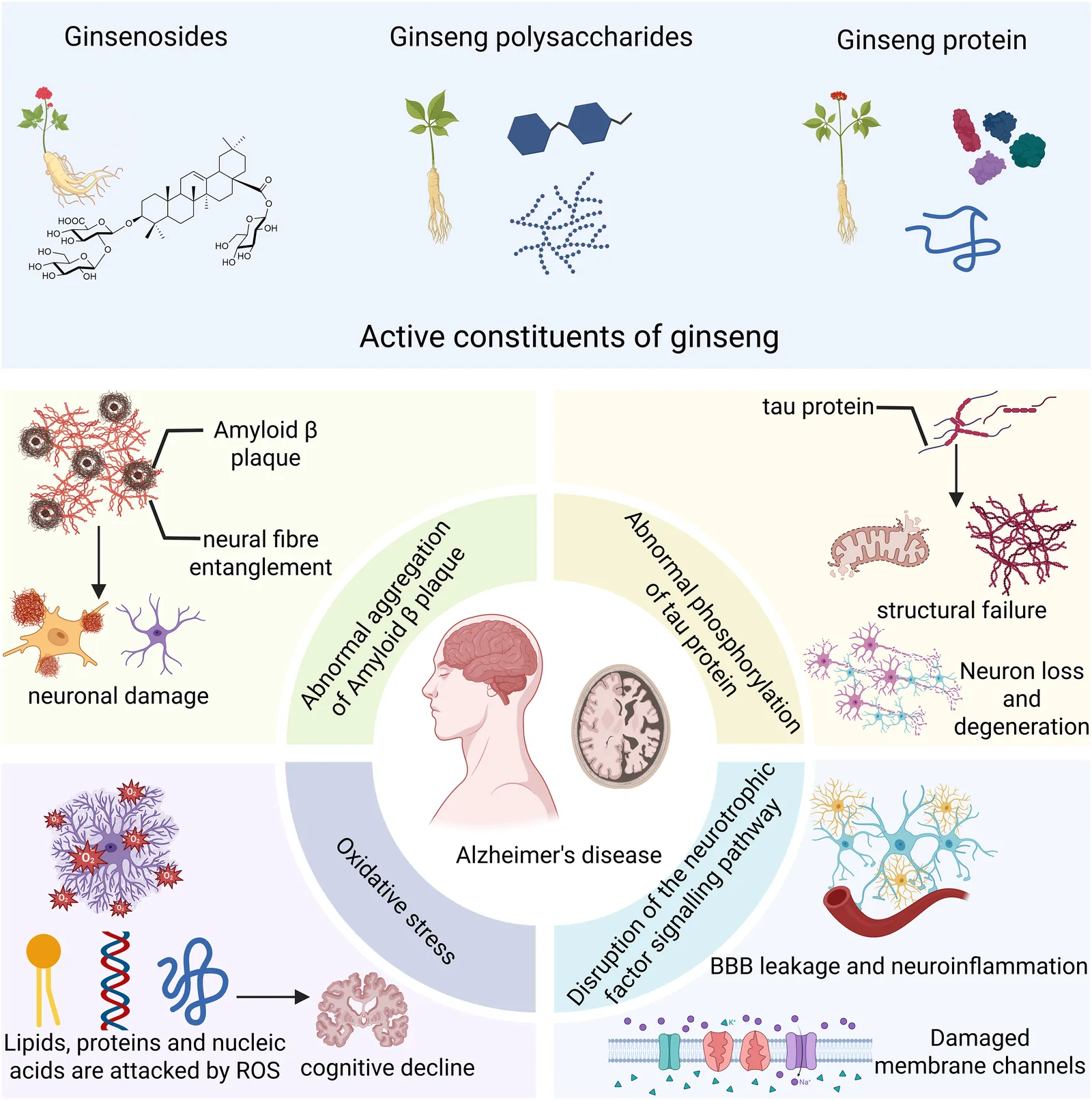
**The active components of ginseng and the pathogenesis of AD.** The active components of ginseng primarily include ginsenosides, ginseng polysaccharides, and ginseng proteins. The pathogenesis of AD mainly involves abnormal accumulation of β-amyloid plaques, abnormal phosphorylation of tau protein, oxidative stress, and disruption of neurotrophic factor pathways.

In summary, AD is a complex neurodegenerative disorder traditionally managed with symptomatic treatments that do not alter disease progression [17]. Neurotrophic factors are increasingly recognized for sustaining neuronal survival and function, showing promise for AD, but their clinical application faces challenges: difficulty in effective brain delivery, low bioavailability and susceptibility to degradation, and limitations regarding treatment timing and patient populations [18]. Ginseng, with its rich bioactive compounds and multi-targeted mechanisms, addresses multiple core AD pathologies, offering novel insights and potential therapeutic strategies [19]. This paper systematically examines the relationship between ginseng and AD treatment, focusing on the correlation between neurotrophic factors and AD. It analyzes the molecular mechanisms by which ginseng targets neurotrophic factors to mitigate neuronal death and dysfunction and outlines future research prospects and strategies to provide theoretical and practical guidance for AD drug development and clinical application. This review presents the following unique innovations: 1. It focuses on the specific regulation of neurotrophic factors by active components of ginseng and their direct association with the pathological features of AD, elucidating the mechanism of their multi-target synergistic action. 2. Unlike previous studies on ginseng, it systematically integrates the gut-brain axis mechanism with multi-omics technologies, proposing a concrete research paradigm for future studies. 3. It integrates traditional medicine, neuropharmacology, nanotechnology, and multi-omics to form an interdisciplinary research strategy. 4. It explicitly addresses key barriers to clinical translation and proposes targeted strategies to overcome them. Through these innovations, this paper offers a more in-depth and translation-oriented perspective on ginseng research in the field of AD (Fig. 1).

## 2 Literature retrieval methods

**Fig. 2:**
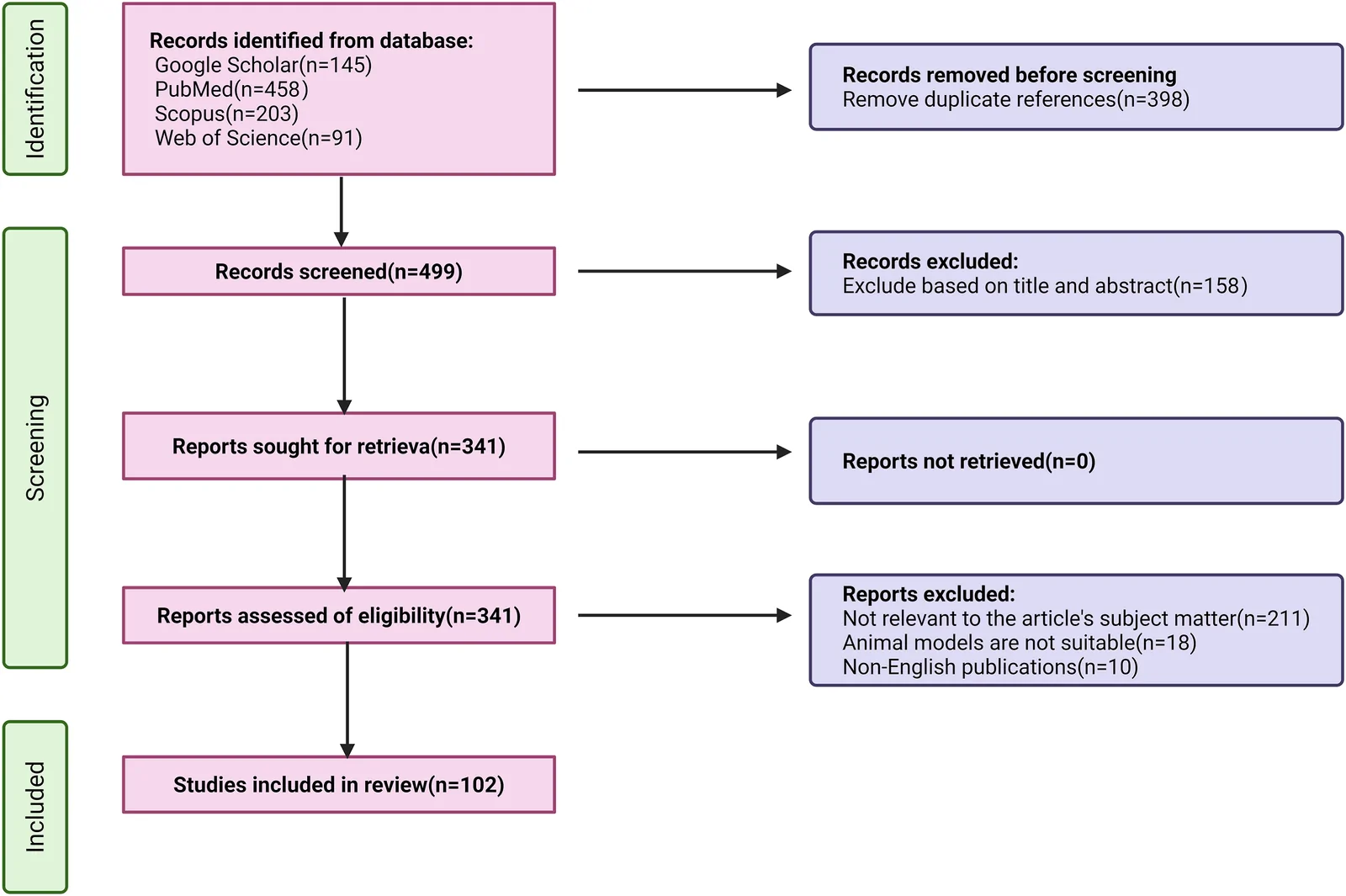
PRISMA flowchart for literature screening.

The core focus of this paper lies in examining the application of ginseng's primary active components and the neurotrophic factors they regulate in treating neurodegenerative diseases, thereby providing a detailed analysis of potential therapeutic strategies for AD. The review primarily covers relevant studies from the past five years to ensure the timeliness and representativeness of the included research. We searched electronic databases including PubMed, Google Scholar, Scopus, and Web of Science using the following free-text keywords: “ginseng,” “Alzheimer's disease,” “ginsenoside Rg1,” “BDNF,” “GDNF,” “ginseng + Alzheimer's disease,” “neurotrophic factors,” “ginseng + neurotrophic factors,” “ginseng + traditional medicine,” “neurotrophic factors + signaling pathways,” and other free-text keywords as core retrieval strategies. At the same time, additional searches were conducted using keywords such as “PI3K/Akt,” “ERK/CREB,” and “TrkB” in relation to topics such as “neurotrophic factor signaling pathways.” To ensure comprehensiveness and rigor, we combined these free-text terms with relevant MeSH subject headings within PubMed to construct robust retrieval formulas. This approach comprehensively covered relevant literature in the field, enabling us to analyze and select studies with sound arguments and well-developed content. Exclusion criteria included: duplicate citations, titles and abstracts that did not match the article content, failure to locate relevant reports, irrelevance to the article's topic, unsuitable animal models, and non-English publications. The literature screening was conducted independently by multiple researchers. An initial screening was performed based on titles and abstracts to exclude studies that clearly did not meet the inclusion criteria; the remaining studies were then subjected to a full-text review. When researchers disagreed, the issue was resolved through discussion and negotiation; if consensus could not be reached, the corresponding author was invited to make the final decision. EndNote was employed for literature synthesis and summarization, while Biorender was utilized for graphical representation. This process culminated in the completion of this review article. (Fig. 2).

## 3 The relationship between ginseng and the treatment of AD

Ginseng contains multiple active constituents—ginsenosides, polysaccharides, and proteins—which possess immunomodulatory, antioxidant, blood-brain barrier permeability-enhancing, and analgesic bioactivities [20]. Ginseng shows significant therapeutic potential in AD by regulating cholinergic function, modulating synaptic plasticity, inhibiting β-amyloid deposition and tau hyperphosphorylation, and counteracting oxidative stress, making it well-suited for complex multifactorial diseases like AD [21].

### 3.1 The correlation between the modern pharmacological effects of ginseng and AD

With advances in modern pharmacology and molecular biology, ginseng has gained experimental and clinical support for preventing and treating AD. AD pathology involves **Aβ** deposition, synaptic dysfunction, neuroinflammation, oxidative stress, mitochondrial dysfunction, and impaired autophagy [22]. Ginseng and its active constituents act on these processes: they markedly inhibit **Aβ** production and aggregation while promoting its clearance; and by modulating the GSK-3β/PP-2A balance, they effectively inhibit tau hyperphosphorylation at multiple sites. This provides novel insights and evidence for AD prevention and treatment [23].

(1) Antioxidant action

Oxidative stress is a central pathological mechanism in the onset and progression of AD; its sources are diverse and give rise to a self-reinforcing vicious cycle. Aβ can aggregate within the inner mitochondrial membrane and matrix, directly inhibiting the activity of complex IV of the electron transport chain, leading to excessive ROS production; simultaneously, the interaction between Aβ and the mitochondrial protein ABAD further exacerbates oxidative stress. Hyperphosphorylated tau protein impedes mitochondrial axonal transport, resulting in abnormal distribution of mitochondria within synapses and cells, and inducing localized energy crises and ROS accumulation. [24]. More importantly, abnormal accumulation of metal ions such as iron and copper in the brains of AD patients generates large amounts of hydroxyl radicals via the Fenton reaction, causing irreversible damage to lipids, proteins, and nucleic acids. These mechanisms are intricately intertwined, making oxidative stress not only an early event in AD but a process that persists throughout the entire course of the disease [25].

Based on this complex network, current evidence suggests that ginsenoside Rg1 and ginsenoside Rb1 may exert their antioxidant protective effects through a three-tiered integrated framework involving “upstream source inhibition—core signaling activation—downstream damage prevention” [26,27]. Among these, the activation of the Nrf2/HO-1 pathway serves as a common core node for both compounds; However, there are significant differences in their mechanisms of action and target molecules. Both ginsenoside Rg1 and ginsenoside Rb1 promote the dissociation of Nrf2 from Keap1 and its translocation into the nucleus, thereby initiating the transcription of antioxidant enzymes such as HO-1, SOD, CAT, and GPx, as well as phase II detoxification enzymes, and enhancing overall antioxidant capacity [15,28]. There are also differences in their antioxidant mechanisms: Ginsenoside Rg1 focuses more on mitochondrial quality control and the remodeling of energy metabolism: it directly stabilizes the mitochondrial membrane potential and optimizes the function of the electron transport chain, thereby reducing ROS production at the source [27]. At the same time, by activating the PI3K/Akt and SIRT1/PGC-1α pathways, it indirectly enhances antioxidant defense and promotes energy metabolism balance [29]. Ginsenoside Rb1, on the other hand, primarily inhibits the activity of enzymes that generate ROS: it suppresses ROS production at the source under pathological conditions by downregulating NADPH oxidase subtypes (NOX-1, -2, and -4) [30]. It also activates the Nrf2/ARE pathway to upregulate HO-1 expression, reduce levels of the lipid peroxidation end product MDA, and inhibit oxidative stress-related apoptotic pathways [31].

The following key research gaps currently exist: all mechanistic inferences are derived from correlational data (changes in biochemical markers), and there is a lack of causal validation through the use of Nrf2 inhibitors, NOX gene knockouts, or targeted antioxidants; The antioxidant effects of ginsenosides Rg1 and Rb1 have mostly been studied in isolation and have not yet been integrated into the “oxidative stress → mitochondrial damage → neuroinflammation” positive feedback loop for comprehensive validation. Furthermore, no studies have simultaneously blocked both the Nrf2 and NOX pathways to assess whether they exhibit synergistic effects. Therefore, conditional knockout mice can be used in the future to conduct causal validation and evaluate the results of the combination therapy of ginsenosides Rg1 and Rb1. Concurrently, optimization and biomarker studies should be conducted to ensure the accuracy of antioxidant research.

(2) Anti-inflammatory effect

Neuroinflammation is a key pathogenic factor driving the progression of AD. Through complex molecular signaling pathways, it directly induces and exacerbates neurotoxicity, promoting neuronal death and cognitive impairment. In the brains of AD patients, pathological proteins such as Aβ plaques and tau tangles continuously activate immune cells in the brain, including microglia and astrocytes. Under physiological conditions, these cells are responsible for clearing metabolic waste, providing neurotrophic support, and maintaining homeostasis; however, in AD patients, they adopt a pro-inflammatory, neurotoxic phenotype [32]. Long-term exposure to Aβ leads to chronic activation of microglia and a loss of responsiveness, resulting in the massive release of pro-inflammatory cytokines such as TNF-α, IL-1β, and IL-6, which directly damage neurons and disrupt synaptic function. At the same time, the ability of microglia to clear Aβ is impaired in AD patients, causing them to accumulate around plaques without being able to effectively phagocytose and degrade Aβ; instead, they continue to secrete inflammatory mediators, thereby exacerbating neuroinflammation [33].

Current evidence suggests that ginsenoside Rg3, ginsenoside Rh2, and ginseng polysaccharides exert anti-inflammatory and protective effects through an integrated mechanism involving “inhibition of microglial activation—regulation of cytokine balance—promotion of neurotrophic signaling.” While the inhibition of pro-inflammatory cytokine release is a common feature among these three compounds, there are significant differences in their mechanisms of action and target molecules. Their shared mechanism of action involves reducing the levels of pro-inflammatory cytokines and improving the inflammatory microenvironment [34,35]. There are also differences in the anti-inflammatory mechanisms of the three: Ginsenoside Rg3 focuses more on directly inhibiting the excessive activation of microglia: by downregulating the expression and release of various pro-inflammatory factors, it directly alleviates inflammation-induced neurotoxicity and provides neurons with a healthier microenvironment [36]. On the other hand, ginsenoside Rh2 exerts a dual neuroprotective effect by directly regulating neurotrophic factor signaling pathways. Specifically, Rh2 significantly upregulates BDNF expression and activates the downstream ERK-CREB signaling pathway, thereby maintaining the normal ERK-CREB-BDNF transcriptional regulatory axis. This mechanism is particularly critical in the context of AD: elevated BDNF levels not only directly counteract synaptic plasticity impairment and neuronal apoptosis caused by Aβ deposition but also inhibit the abnormal phosphorylation of tau protein at the Ser396 site via the ERK pathway, thereby simultaneously addressing two core pathological features of AD. Furthermore, the ERK-CREB-BDNF signaling homeostasis maintained by ginsenoside Rh2 helps enhance neuronal tolerance to inflammatory stress, thereby indirectly reducing the release of pro-inflammatory cytokines. Therefore, the dual effects of ginsenoside Rh2—“neurotrophic signaling and anti-inflammation”—are not two independent pharmacological actions, but rather a synergistic integration achieved through the ERK-CREB-BDNF signaling axis: BDNF itself inhibits excessive microglial activation and reduces IL-1β and TNF-α levels. This mechanism closely links Rh2's neurotrophic factor regulation to the neuroinflammation, synaptic loss, and tau pathology associated with AD, highlighting its therapeutic potential to counteract multiple pathological aspects of AD by strengthening endogenous neurotrophic support pathways [37]. Ginseng polysaccharides primarily focus on reprogramming cytokine balance: by reducing levels of pro-inflammatory cytokines (IL-6, TNF-α) and increasing levels of anti-inflammatory cytokines (IL-10), they improve the inflammatory microenvironment, helping to maintain the integrity of the blood-brain barrier and promote synaptic plasticity [38]. These effects collectively mitigate the neuronal damage and neuroinflammation-driven vicious cycle in AD, thereby helping to maintain the integrity of the blood-brain barrier and promote synaptic plasticity.

Based on the anti-inflammatory mechanisms, the following key research gaps currently remain: Current studies only measure overall levels of pro-inflammatory factors and do not distinguish between the M1 and M2 polarization states of microglia. This is critical in AD treatment, as completely inhibiting microglia may interfere with their physiological function of clearing Aβ. In addition, ginsenoside Rg3, ginsenoside Rh2, and ginseng polysaccharides naturally coexist in ginseng; however, no studies have yet evaluated whether these three components exhibit synergistic or antagonistic effects. Therefore, in the future, it will be possible to use mice with conditional knockout of NLRP3 or BDNF specifically in microglia to conduct causal validation studies. Additionally, we can systematically compare the dose-response relationships and combination therapy of ginsenoside Rg3, ginsenoside Rh2, and ginseng polysaccharides in the same AD model, while dynamically monitoring changes in microglial morphology and Aβ plaque formation.

(3) Neuroprotection and synaptic function improvement

Ginseng and its bioactive components exert neuroprotective effects and improve synaptic function by establishing an integrated four-tier framework comprising “antioxidant defense—glutamate homeostasis—epigenetic regulation—mitochondrial support”. Compound K (CK) is the primary active metabolite of ginsenosides in the body. It exerts antioxidant protection through direct antioxidant activity and activation of the Nrf2 pathway: on the one hand, it directly scavenges excess ROS and RNS, thereby reducing oxidative stress-induced damage to neuronal membrane lipids, proteins, and DNA; on the other hand, it activates the Nrf2/ARE signaling pathway—the primary regulator of the cellular antioxidant response—promoting the expression of various downstream antioxidant enzymes and enhancing the intrinsic antioxidant capacity of neurons [39]. CK can also alleviate excitotoxic damage caused by excessive glutamate stimulation: it antagonizes the excessive activation of NMDA receptors while promoting glutamate reuptake (by enhancing EAAT2 function in astrocytes), thereby maintaining glutamate homeostasis and exerting a neuroprotective effect [40]. miR-9-5p is a microRNA specifically expressed in the brain and closely associated with AD pathology. In AD, its expression levels are abnormally elevated. Ginsenoside Rg1 effectively downregulates the overexpression of miR-9-5p while simultaneously upregulating the expression and activity of the SIRT1 protein. SIRT1 is an NAD + -dependent deacetylase and a key regulator of cellular energy metabolism and mitochondrial function. Increased expression of this enzyme improves mitochondrial function and exerts neuroprotective effects [41]. Furthermore, by alleviating synaptic toxicity caused by mitochondrial dysfunction, it maintains synaptic structure and number, reduces synaptic damage, and enhances synaptic function [42].

Based on the above, the following key research gaps remain: CK is an in vivo metabolite of various saponins in humans, but no studies have systematically evaluated the relative potencies of CK and its precursor saponins. There is a lack of PK/PD-based therapeutic window studies, which prevents the support of dose conversion from animals to humans. Additionally, there are no clinical studies in AD patients that have validated cerebrospinal fluid levels of SIRT1, miR-9-5p, or microglial polarization markers as biomarkers for ginseng's effects. Therefore, future research should include comparative pharmacodynamic studies of CK and protosaponins to elucidate the relationship between in vivo metabolism and biological activity; Golgi staining and electrophysiological methods should be employed to directly assess synaptic structure and function. More importantly, clinical studies on biomarkers in the cerebrospinal fluid of AD patients should be conducted.

(4) Improving the function of the cholinergic system

Ginsenosides systematically improve cholinergic dysfunction in AD by establishing a three-tiered integrated framework that involves “reducing degradation, promoting synthesis, and enhancing signal transduction.” Acetylcholinesterase is the key enzyme responsible for breaking down acetylcholine in the brain. Ginsenosides inhibit the activity of this enzyme, thereby reducing the degradation of acetylcholine, increasing its concentration in the synaptic cleft, and improving cholinergic neurotransmission. Studies have shown that various ginsenosides exhibit significant inhibitory effects on acetylcholinesterase [43]. Ginsenosides not only **reduce** the breakdown of acetylcholine but also promotes its synthesis. Choline acetyltransferase (ChAT) is the key enzyme responsible for synthesizing acetylcholine (AChE), and its activity is typically reduced in the brains of AD patients. Ginsenosides exert their effects in enhancing cholinergic system function by regulating the expression of choline acetyl transferase [44]. Acetylcholine must bind to receptors (M-type cholinergic receptors and N-type nicotinic receptors) to exert its effects. Various ginsenosides enhance the efficiency of cholinergic signaling by regulating the expression and function of these receptors, enabling acetylcholine to exert its effects more effectively after release. This mechanism effectively restores acetylcholine levels and function, thereby reversing the cholinergic dysfunction and memory impairment caused by AD [45,46].

Based on the above, the following key research gaps currently exist: No studies have compared the AChE inhibitory potency, ChAT upregulation capacity, and receptor subtype selectivity of various ginsenosides under the same experimental conditions. At the same time, it has not been evaluated whether long-term inhibition of AChE leads to receptor desensitization or compensatory upregulation. The lack of comparative studies with first-line AD treatments makes it impossible to assess the relative efficacy or adjunctive value of ginsenosides.

Therefore, future studies should employ brain microanalysis techniques to simultaneously measure AChE activity, ACh concentration, and behavioral changes in specific brain regions, thereby establishing a comprehensive relationship between drug efficacy and cognitive function and assessing whether receptor desensitization or compensatory enzyme upregulation occurs. Concurrently, preclinical studies should be designed to evaluate the effects of ginsenosides when used alone or in combination with first-line AD medications.

(5) Anti-apoptotic effect

Neuronal apoptosis is one of the key factors contributing to cognitive decline. Ginsenosides exert their anti-apoptotic effects by establishing a three-tiered integrated framework that involves regulating BCL-2 family protein interactions, blocking the mitochondrial apoptosis pathway, and intervening in the pathological vicious cycle of Aβ/tau. In AD, neuronal apoptosis is one of the key factors contributing to cognitive decline, with the apoptotic process being regulated by BCL-2 family proteins. Overexpression of anti-apoptotic proteins (such as BCL-2, BCL-XL, and MCL-1) inhibits neuronal apoptosis; however, in AD, their dysfunction may lead to abnormal cell survival or signaling disruption. Concurrently, pro-apoptotic proteins promote apoptosis [47]. Concurrently, cytochrome C released into the cytoplasm forms apoptosomes with proteins such as Apaf-1, thereby activating initiator caspases (caspase-9). Caspase-9 subsequently activates effector caspases (caspase-3, -6). Activated executive caspases cleave multiple critical cellular proteins, leading to the morphological hallmarks of apoptosis, commonly observed as cell shrinkage and nuclear condensation. The literature indicates that activated Caspase-3 is detected in the brains of AD patients, and it can cleave both APP and tau proteins, thereby exacerbating Aβ production and tangling formation, creating a vicious cycle [48]. Ginsenosides Rg3, Rh2, and Rf bind to BCL-2, BCL-XL, and MCL-1, thereby blocking their interaction with pro-apoptotic proteins. This restores the apoptotic balance, facilitating the clearance of damaged or dysfunctional neurons. Different ginsenosides exhibit selective binding capacity towards distinct anti-apoptotic proteins, suggesting that ginseng may exert synergistic anti-apoptotic effects through a multi-component, multi-target mechanism. Molecular dynamics simulations indicate that the binding structure between ginsenosides and anti-apoptotic proteins is stable, with hydrogen bonds and van der Waals forces constituting the primary interactions. This suggests they may exhibit sustained biological activity in vivo, providing novel evidence for their therapeutic potential in treating AD [49].

Based on the above, the following key research gaps currently exist: there is a lack of studies directly measuring the activity of caspase-9 and caspase-3/6 in AD cell and animal models to verify the actual inhibitory effects of saponin treatment. Additionally, research on the mitochondrial apoptosis pathway should include the detection of mitochondrial cytochrome C release, mitochondrial membrane potential (JC-1/TMRM), and the formation of apoptosomes. There is a lack of cell-type-specific studies examining whether the anti-apoptotic effects are consistent across neurons, astrocytes, and microglia. Therefore, future studies should validate the inhibitory effects of ginsenosides on caspase activity and apoptosis rates in AD cell models (primary neurons treated with Aβ). At the same time, the specificity of the ginsenoside effects should be verified using a selective BCL-2 inhibitor.

### 3.2 Multi-target and multi-pathway regulatory characteristics of ginseng bioactive components

Ginseng's pharmacological effects derive from its diverse active constituents (e.g., ginsenosides and polysaccharides), whose multi-targeted, multi-pathway synergistic actions confer unique therapeutic advantages in managing complex diseases such as AD.

(1) Ginsenosides

Ginsenosides are the primary active constituents of ginseng, with over 100 identified compounds. Based on aglycone structures, they are classified into protopanaxadiol-type (PPD), protopanaxatriol-type (PPT), and oleanolic acid-type (OA). Structural modifications can alter bioactivity: reducing sugar moieties enhances lipophilicity; modifying the C-20 stereo configuration confers distinct biological activities and pharmacokinetic properties; dehydration and epoxidation increase bioactivity [50].

Different ginsenosides act via distinct mechanisms in AD. Ginsenoside Rg1 inhibits the IL-17 signaling pathway to reduce pro-inflammatory factors, thereby suppressing neuroinflammation. In lipid and atherosclerosis pathways—relevant to lipid metabolism disorders in the AD brain—Rg1 helps reduce lipid peroxidation and protects neurons. The AGE-RAGE pathway exacerbates oxidative stress and inflammation in AD; Rg1 may interrupt end-product accumulation to alleviate symptoms [51]. Additionally, Rg1 corrects mitochondrial fission/fusion imbalance by activating AMPK and inhibiting excessive Drp1 activation, improving mitochondrial function and exerting neuroprotection against AD [52].

Ginsenoside Rb1 exerts neuroprotection by activating PPARγ (a nuclear receptor regulating energy metabolism, inflammation, and cell survival). Rb1 also promotes intracellular cholesterol efflux, reducing cholesterol levels, which alleviates neuroinflammation, protects neurons, alleviates AD symptoms, and promotes nervous system repair [53]. In AD, APP is primarily cleaved by β- and γ-secretases via the amyloidogenic pathway, producing toxic Aβ peptides and plaques. Rb1 redirects APP cleavage from the amyloidogenic to the non-amyloidogenic pathway, reducing Aβ production at its source, thereby decreasing hippocampal Aβ plaque deposition and improving learning and memory in AD [54].

Ginsenoside Re has potent antioxidant effects in AD treatment. Under oxidative stress, Re promotes Nrf2 translocation from cytoplasm to nucleus, where it binds to the antioxidant response element, initiating expression of downstream antioxidant genes including heme oxygenase-1, quinone reductase 1, and glutathione S-transferase catalytic subunit. This upregulation increases intracellular glutathione levels and enhances activity of antioxidant enzymes such as superoxide dismutase and glutathione peroxidase, thereby counteracting oxidative stress and neuronal damage in AD [55].

Ginsenoside Rh2 exerts multiple effects in AD treatment. It modulates cholinergic transmission by inhibiting acetylcholinesterase activity, reducing acetylcholine breakdown and increasing brain acetylcholine levels, thereby addressing cholinergic deficits and improving learning and memory. Concurrently, Rh2 exerts antioxidant effects by enhancing superoxide dismutase and glutathione peroxidase activity and reducing lipid peroxide levels, mitigating oxidative stress-induced neuronal damage and protecting neuronal membrane integrity and functional structures [37].

(2) Ginseng polysaccharides

Ginseng polysaccharides play significant roles in immunomodulation and neuroprotection. A 4.7-kDa polysaccharide (GP4) purified from ginseng inhibits Aβ aggregation and pathological manifestations across multiple AD models by activating mitochondrial autophagy, while also ameliorating memory and cognitive deficits [56]. In AD, hyperactivated microglia release inflammatory mediators that exacerbate neuronal damage. Ginseng polysaccharides inhibit excessive microglial activation, downregulate pro-inflammatory cytokines (TNF-α, IL-6), and promote anti-inflammatory IL-10, creating an anti-inflammatory brain environment and protecting neurons. They also protect mitochondrial function, ensuring energy supply and neuronal health [57]. Additionally, ginseng polysaccharides activate the Keap-1/Nrf2 signaling pathway: they disrupt Keap-1-Nrf2 binding, preventing Nrf2 degradation; free Nrf2 translocates to the nucleus, binds to the antioxidant response element (ARE), and initiates transcription of protective genes. This mitigates oxidative damage, suppresses neuroinflammation, reduces neuronal death, and alleviates AD symptoms [58].

**Fig. 3:**
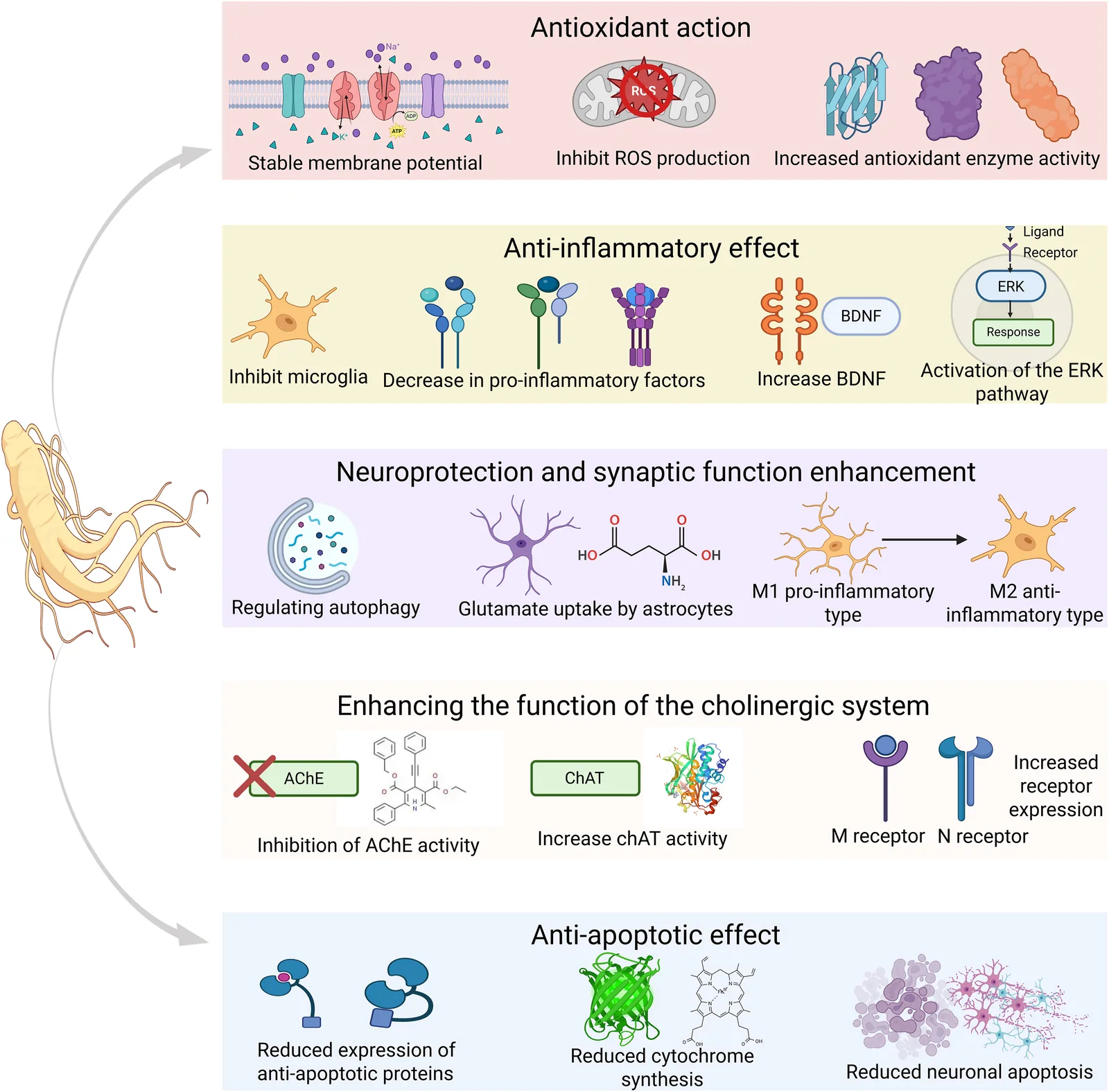
**Modern pharmacological effects of ginseng and its relevance to AD.** The modern pharmacological effects of ginseng primarily include antioxidant activity, anti-inflammatory effects, neuroprotection and synaptic function enhancement, cholinergic system function improvement, and anti-apoptotic effects. These mechanisms collectively contribute to alleviating symptoms of AD.

In summary, ginseng's active constituents exert multifaceted regulatory effects via multiple targets and pathways, collectively intervening in AD pathology. Ginsenosides primarily focus on directly influencing neuronal function and pathological protein deposition, while ginseng polysaccharides predominantly exert immunomodulatory and anti-inflammatory effects. This dual action renders ginseng a valuable natural treasure trove for AD therapeutic research (Fig. 3).

## 4 The correlation between neurotrophic factor signaling pathways and AD

Neurotrophic factor signaling pathways are crucial for neuronal survival, synaptic plasticity, and cognition. In AD, Aβ deposition and tau abnormalities impair these pathways, causing weakened signaling, synaptic loss, and neuronal death, leading to cognitive impairment. Hence, enhancing this signaling is a potential therapeutic strategy for AD.

### 4.1 Alterations in neurotrophic factors in the pathology of AD

**Fig. 4:**
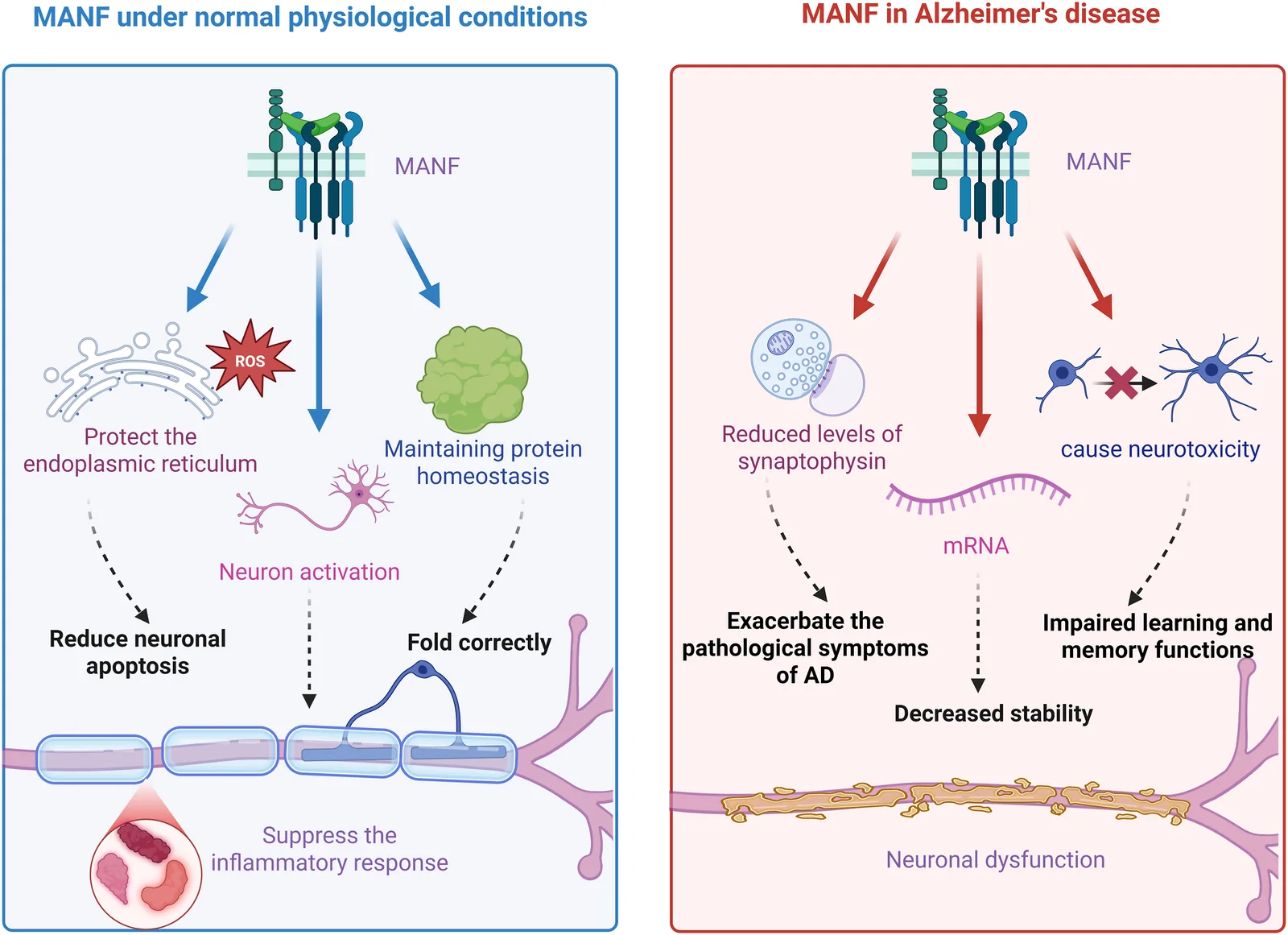
MANF **under normal physiological conditions and MANF in AD.** Under normal physiological conditions, MANF regulates endoplasmic reticulum oxidative stress and maintains protein homeostasis, while reducing neuronal apoptosis and suppressing inflammation. In the pathological state of AD, high expression of MANF promotes the secretion of synaptophysin, leading to cytotoxicity and ultimately exacerbating neuronal damage in AD patients.

Neurotrophic factors are proteins crucial for neuronal survival, differentiation, synaptic plasticity, and functional maintenance. In AD, multiple neurotrophic factors exhibit altered expression, signaling, or receptor systems, closely linked to cognitive impairment and neurodegeneration. NGF expression is upregulated in AD brains and cerebrospinal fluid, yet its high-affinity receptor TrkA is downregulated (particularly in cortex and basal ganglia), leading to cholinergic terminal degeneration and abnormal non-amyloid APP processing [59]. BDNF is essential for synaptic structure, function, and long-term potentiation (LTP), the cellular basis of learning and memory. In AD brains, BDNF mRNA is significantly reduced, directly causing synaptic loss and dysfunction. BDNF also exerts anti-apoptotic effects via downstream TrkB/PI3K/Akt signaling; its deficiency in AD renders neurons more vulnerable to stressors such as Aβ toxicity. Consequently, diminished BDNF signaling leads to cognitive decline, learning and memory impairments, and behavioral abnormalities [60]. Under normal physiological conditions, mesencephalic astrocyte-derived neurotrophic (MANF) regulates endoplasmic reticulum stress and maintains protein homeostasis. The C-terminus of MANF contains a reticulum-targeting signal, which allows it to localize primarily within the lumen of the endoplasmic reticulum. Therefore, it is considered an endoplasmic reticulum-resident protein. Under normal physiological conditions, MANF helps maintain endoplasmic reticulum homeostasis. When endoplasmic reticulum stress occurs, MANF expression is upregulated, assisting in proper protein folding, mitigating the unfolded protein response, and protecting cells from endoplasmic reticulum stress-induced apoptosis. In the nervous system, MANF possesses intrinsic cytoprotective effects. It inhibits the activation of the caspase family under oxidative stress, thereby reducing neuronal apoptosis [61,62]. Furthermore, MANF participates in the proliferation, differentiation, and maturation of neural progenitor cells and contributes to the maintenance of immune cell homeostasis. More importantly, under normal physiological conditions, it regulates the polarization of macrophages and astrocytes, suppresses excessive inflammatory responses, and promotes tissue repair [63]. The expression level of MANF is significantly elevated in hippocampal tissue from AD patients. Concurrently, levels of synaptophysin were significantly reduced. In AD, the sustained, high endogenous expression of MANF within cells exerted neurotoxic effects, directly leading to synaptic loss and impaired learning and memory. MANF directly interacts with the RNA-binding protein ELAVL2 in AD patients, with the binding site being the third RNA recognition motif (RRM3) of ELAVL2. Overexpression of MANF enhances ELAVL2's binding affinity for multiple mRNAs. This abnormal increase in binding disrupts the stability of mRNAs critical for synaptic function, leading to dysregulated expression levels and exacerbating AD symptoms (Fig. 4) [64]. p75NTR is widely expressed in the developing brain, with its expression narrowing in adulthood to basal forebrain cholinergic neurons (BFCNs) and hippocampal CA1 neurons/astrocytes. In AD, BFCNs degenerate early and most severely, heralding the spread of AD neuropathology. Concurrently, p75NTR promotes Aβ production and mediates its downstream toxicity, exacerbating synaptic loss and cognitive impairment. p75NTR signaling also promotes tau hyperphosphorylation, further exacerbating neurodegeneration [65]. **Neurotrophin-4/5 (NT-4/5)** typically exhibit reduced protein and mRNA levels in the brains of AD patients, particularly within regions associated with learning and memory such as the hippocampus and cortex. This decline is frequently correlated with the severity of AD pathology and the degree of cognitive decline. NT-4/5 exerts crucial protective effects in AD by binding to its high-affinity receptor TrkB, thereby activating multiple key intracellular signaling pathways. These effects include promoting neuronal survival, enhancing synaptic plasticity and cognitive function, and maintaining synaptic structure and function. However, reduced expression levels in AD patients lead to neuronal vulnerability and cognitive decline, thereby accelerating disease progression (Table 1) [76].

**Table 1:** The neuroprotective mechanism of ginseng's active components in AD is mediated through the regulation of neurotrophic factors.

Active constituents of ginseng	Primarily targets neurotrophic factors	Signaling pathways	Experimental model	Primary Pharmacological Mechanism of Action
Ginsenoside Rg1	BDNF	TrkB/PI3K/Akt	APP/PS1 mice, Aβ25–35 induces rats	Ginsenoside Rg1 can reduce the production of Aβ and promote its clearance, thereby alleviating the neuronal toxicity and inflammatory response triggered by Aβ accumulation within the brain. Concurrently, ginsenoside Rg1 inhibits the kinase activity responsible for abnormal tau protein phosphorylation, thereby aiding in the maintenance of neuronal cytoskeletal stability. Furthermore, ginsenoside Rg1 enhances BDNF expression, improves LTP and spatial memory, and inhibits neuronal apoptosis [66,67].
Ginsenoside Rb1	NGF	JNK/ERK/NF-κB	PC12 cells, AD transgenic mice	Ginsenoside Rb1 downregulates the activity of the downstream NF-κB signaling pathway by inhibiting the phosphorylation of JNK and ERK. Inhibition of the NF-κB pathway reduces the production and release of pro-inflammatory cytokines, thereby effectively alleviating inflammatory responses within the central nervous system and exerting neuroprotective effects. Concurrently, ginsenoside Rb1 modulates the NDUFS4-SIRT5-DUSP1 axis to coordinate mitochondrial quality control with inflammatory responses, mitigating neurological damage. Moreover, ginsenoside Rb1 promotes NGF secretion and receptor activation, thereby enhancing cholinergic function [[68], [69], [70]].
Ginsenoside Re	NGF, BDNF	NLRP3/PINK1	C57BL/6J mice	Ginsenoside Re significantly inhibits NLRP3 inflammasome activation, reducing the maturation and release of inflammatory cytokines, thereby alleviating neuroinflammation. By promoting mitochondrial autophagy, it indirectly inhibits NLRP3 inflammasome activation, thereby establishing a virtuous cycle of ‘mitochondrial autophagy—inhibiting inflammation’. Concurrently, ginsenoside Re can eliminate dysfunctional mitochondria by enhancing PINK1-mediated mitochondrial autophagy, thereby reducing the accumulation of ROS and neuronal damage resulting from mitochondrial dysfunction. Additionally, it also promotes neurite outgrowth and enhances synaptic plasticity [71,72].
Ginsenoside Rh2	BDNF/GDNF	pERK1/2/pAKT/pCREB/NF-κB	C57BL/6 mice	Ginsenoside Rh2 can upregulate the expression of BDNF and GDNF proteins in hippocampal tissue, activating downstream signaling pathways including pERK1/2, pAKT, and pCREB. These molecules collectively form a signaling cascade that promotes neuronal survival, synaptic plasticity, and neurogenesis [73].
Ginseng polysaccharides	BDNF	Nrf2**/**HO-1/NF-κB	APP/PS1 transgenic mice, AD transgenic mice	Ginseng polysaccharides activate the Nrf2/HO-1 pathway, thereby upregulating the expression of multiple downstream antioxidant enzymes and phase II detoxification enzymes. This facilitates the clearance of ROS and protects neurons. Concurrently, ginseng polysaccharides mitigate neuroinflammation by inhibiting NF-κB activation, thereby reducing the production of pro-inflammatory cytokines [74,75].

### 4.2 The importance of neurotrophic factors as intervention targets for AD

Based on evidence, neurotrophic factors are considered key therapeutic targets for AD, and intervention strategies involving exogenous supplementation or enhancement of their levels have gained significant attention in recent years.

(1) Gene therapy

Gene therapy is a revolutionary strategy aimed at correcting disease pathology at its root, offering long-term or even permanent treatment. Its core principle involves delivering therapeutic genes into the CNS via viral vectors to counteract AD pathology. Small interfering RNA or antisense oligonucleotides can suppress APP or BACE1 expression, reducing Aβ production at its source. Antisense oligonucleotides can also target MAPT mRNA to reduce tau protein levels, diminishing substrate for neurofibrillary tangles. Protective ApoE2 genes may be introduced to counteract ApoE4 effects, improving lipid metabolism, reducing Aβ aggregation, and mitigating tau pathology [77]. NGF gene delivered via AAV to basal forebrain cholinergic neurons secretes NGF protein, which supports and restores degenerating cholinergic neurons by activating TrkA and p75NTR. BDNF gene delivered to hippocampus and cortex exerts effects via TrkB, enhancing synaptic plasticity, supporting neuronal survival, promoting memory formation, and counteracting synaptic toxicity. Introducing anti-inflammatory cytokine genes (IL-10, TGF-β) suppresses chronic neuroinflammation, improving neural damage and synaptic function in AD patients [78].

(2) Protein delivery

Protein delivery is the most employed intervention strategy. A ‘2 + 1’ bispecific antibody delivery system has distinct advantages: two bivalent binding sites ensure high affinity, and one monovalent binding site for transferrin receptor 1 (TfR1) mediates blood-brain barrier transport, circumventing efficacy limitations. The TfR1-binding module is attached to the Fc fragment's C-terminal end, preventing Fc-mediated dysfunction. Protein delivery enables more uniform brain drug distribution, potentially achieving comprehensive, dead-zone-free clearance of Aβ plaques. It also shows superior efficacy in poorly responsive brain regions such as the medial temporal cortex [79].

(3) Small-molecule agonists and enhancers

Small-molecule agonists and enhancers are a therapeutic strategy for AD that modulates intrinsic physiological processes, providing neuroprotection and disease modification, complementing symptomatic therapies and biologics. In AD, cholinergic neurotransmission is impaired, especially in learning and memory regions. The M1 receptor, highly expressed in these areas, regulates cognitive function. The M1 agonist HTL9936 shows effective activation in central regions with high M1 expression but reduced activity in peripheral areas with low expression, offering diminished side effects. It is highly selective for M1 (scarcely activating M2/M3/M5), reverses memory deficits, enhances cognition, activates memory-related brain regions, and has favorable safety [80]. As a partial agonist of TrkB and TrkC, TX-BD10-2 (BD10-2) restores hippocampal LTP deficits, improving synaptic function and plasticity. It promotes synaptic structure and protein expression, increasing excitatory synapses. BD10-2 also restores LTP-associated activity-dependent gene expression, enhancing immune- and microglia-related genes, thus regulating transcriptome expression. Compared to its parent compound LM22B-10, BD10-2 has superior oral bioavailability and brain distribution, activates both TrkB and TrkC, mimics neuroprotective effects of BDNF and NT-3, and shows no significant adverse effects at therapeutic doses. It does not activate TrkA or p75, indicating high receptor selectivity [81].

**Fig. 5:**
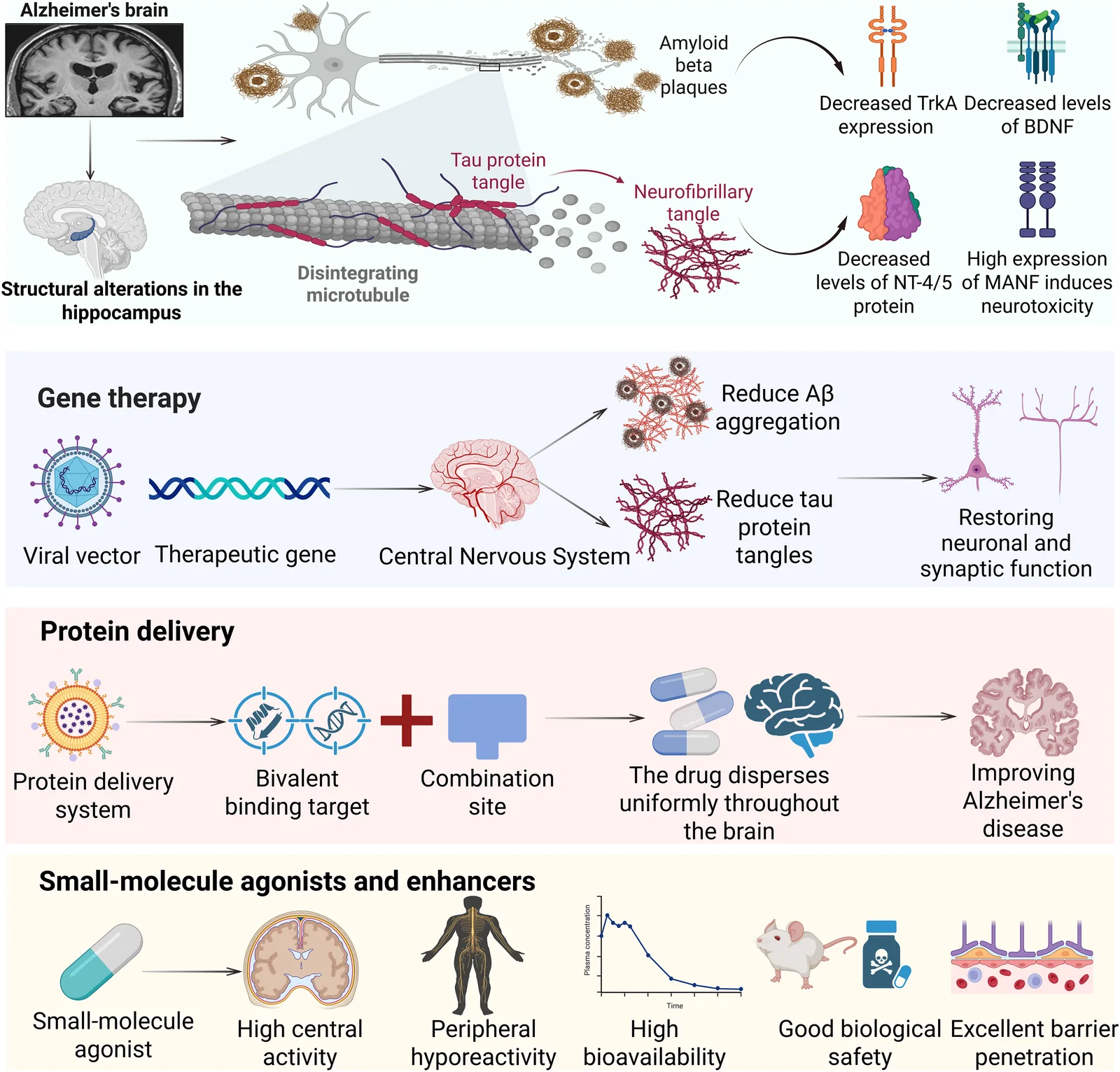
**Changes in neurotrophic factors in AD pathology and intervention methods and strategies for AD.** The hallmark pathology of AD (Aβ plaque deposition and tau protein tangles) can lead to reduced TrkA protein expression, decreased levels of brain-derived neurotrophic factor, elevated MANF expression, and decreased expression of neurotrophic factors 4/5. Intervention methods and strategies for AD primarily include gene therapy, protein delivery, and the use of small-molecule agonists and enhancers.

In summary, neurotrophic factors play a central role in AD pathogenesis and progression, with reduced levels and impaired signaling driving cognitive impairment. Intervention strategies targeting these factors—particularly multi-target regulatory approaches combined with natural medicines such as ginseng active components—may offer novel avenues for AD treatment (Fig. 5).

## 5 Outlook and research strategy

AD's complexity limits single-mechanism drugs. Ginseng, as a natural multi-targeted agent, shows potential via regulating neurotrophic factor networks, but clinical application requires further breakthroughs in mechanism research and formulation development. This section explores two key future research directions.

### 5.1 Deepening research into mechanisms

(1) Multi-omics strategies reveal comprehensive functional networks

**Fig. 6:**
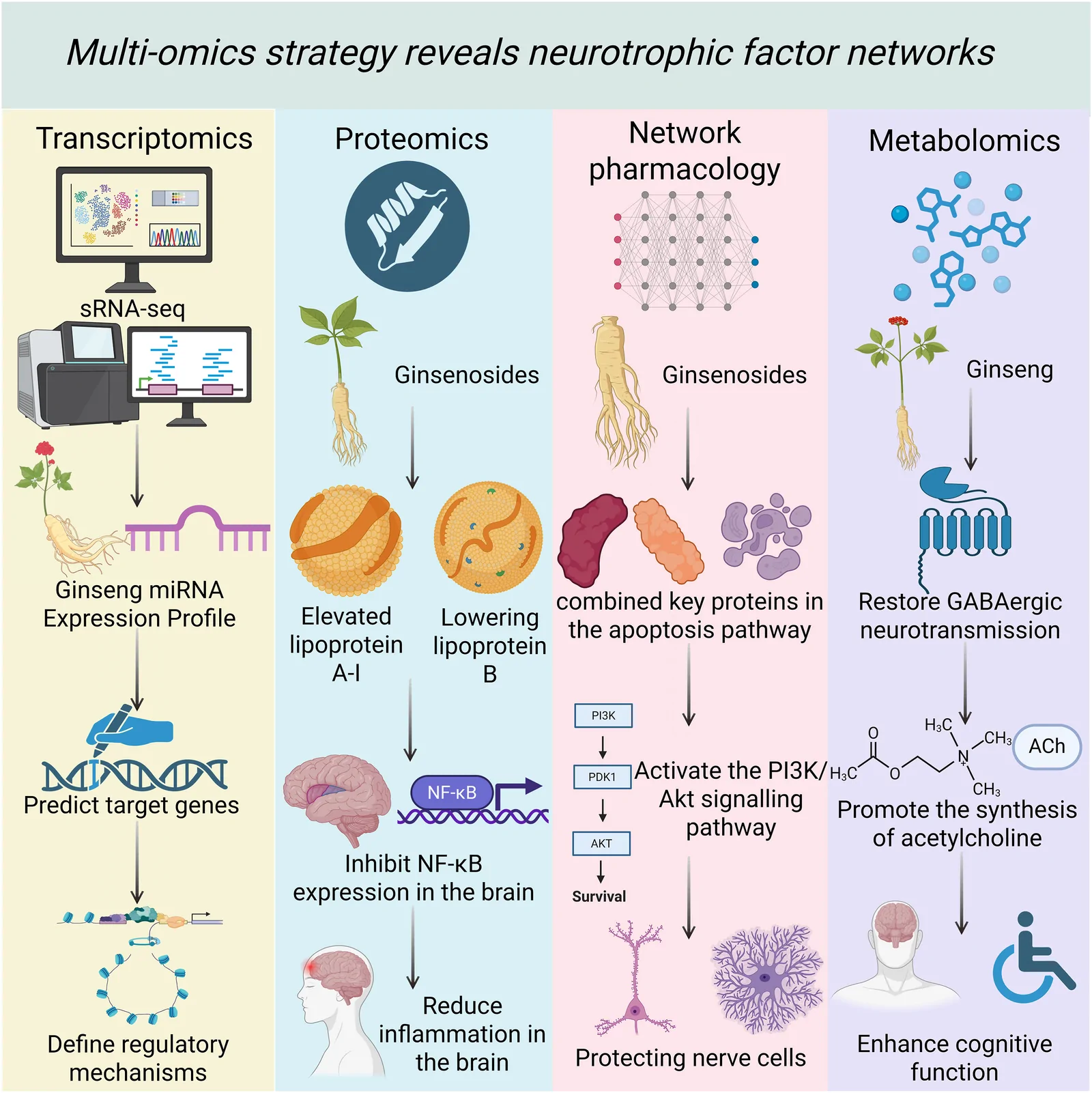
**Multi-omics strategies reveal neurotrophic factor networks.** These strategies primarily encompass transcriptomics, proteomics, network pharmacology, and metabolomics. Transcriptomics utilizes sRNA-seq to analyze ginseng miRNA expression profiles, predict target gene functions, and elucidate ginseng's regulatory networks. Proteomics reveals that ginsenosides upregulate apolipoprotein A-I, downregulate apolipoprotein B, inhibit NF-κB expression, and alleviate brain inflammation. Network pharmacology elucidates interactions between ginsenosides and key proteins in the apoptosis pathway, reducing neuronal cell death by inhibiting anti-apoptotic proteins and activating the PI3K/Akt pathway. Metabolomics reveals that ginseng restores GABAergic neurotransmission function, promotes acetylcholine synthesis, and improves AD-related cognitive impairment.

Future research should employ multi-omics integrated analysis (transcriptomics, proteomics, network pharmacology, metabolomics) to elucidate ginseng's systemic regulation of the neurotrophic factor network [82]. Transcriptomics via sRNA-seq identifies miRNA profiles in ginseng roots, predicting target genes involved in ginsenoside biosynthesis [83].Proteomics shows that ginsenoside Rb1 corrects lipid metabolism disorders by upregulating apolipoprotein A-I and downregulating apolipoprotein B, while suppressing NF-κB pathway proteins in prefrontal cortex and hippocampus, downregulating pro-inflammatory cytokines, alleviating neuroinflammation, and promoting recovery in AD [84]. Network pharmacology and proteomics reveal that ginsenoside Re binds strongly to apoptosis key proteins (Caspase-3, BAX, BCL2) and activates PI3K/Akt, an anti-apoptotic survival pathway, reducing neuronal and synaptic death [85]. Metabolomics shows ginseng upregulates succinic acid semialdehyde, a GABA metabolic substrate, helping restore GABAergic transmission, regulate neural activity, activate cerebral glucose metabolism, and promote acetylcholine synthesis, improving cognition [86]. Multi-omics integration will establish a comprehensive network of ginseng, neurotrophic factors, and neuroprotection (Fig. 6).

(2) Exploring the ginseng–gut microbiota–neurotrophic factor axis

The active constituents of ginseng exhibit a bidirectional regulatory relationship with the gut microbiota during their metabolic processes within the body: ginseng and its components can alter the composition of intestinal microorganisms, promote the growth of beneficial bacteria while inhibit harmful ones. Gut microbiota can convert ginseng's protosaponins into rare saponins with enhanced biological activity and improved bioavailability by secreting specific enzymes such as glucosidase (Table 2) [95]. Future research may focus on the tripartite interaction among ginseng, gut microbiota, and neurotrophic factors. Ginseng and its active constituents (ginsenosides, polysaccharides) modulate gut microbiota composition, thereby influencing brain function and exerting neuroprotection. In AD, ginseng improves memory and cognition by regulating gut microbiota, reducing inflammatory factors and Aβ1–42. Concurrently, gut microbiota convert protopanaxadiol saponins into more bioactive metabolites with enhanced anti-inflammatory, antioxidant, and anti-apoptotic effects, alleviating AD symptoms. Ginseng improves D-galactose-induced memory impairment by modulating Bacteroidetes and Lactobacillus and activating the cAMP signaling pathway [96]. Ginsenoside R1 restores gut microbiota, inhibits the brain TLR4/MyD88/NF-κB pathway, reduces microglial and astrocytic activation, suppresses neuroinflammation, and indirectly elevates BDNF and NGF levels, thereby enhancing the BDNF/NGF pathway [97].

**Table 2:** Gut microbiota-mediated conversion of ginseng protosaponins into rare saponins.

Prototype ginsenoside	Rare saponins generated through conversion	Key gut microbiota involved	Key enzymes involved in secretion	Key conversion steps	Changes in properties after conversion
Ginsenoside Rg1	Ginsenoside Rh1	Lactobacillus spp	β-glucosidase	Ginsenoside Rg1 → Ginsenoside Rh1 Removal of a glucose residue at the C-20 position	Molecular weight: reduced Activity: enhanced neuroprotection and anti-vascular endothelial damage [87,88]
Ginsenoside Rb1	Compound K (CK)	Bacteroides spp; Bifidobacterium spp; Lactobacillus spp	β-glucosidase; β-galactosidase	Ginsenoside Rb1 → Ginsenoside Rd → Ginsenoside F2 → CK Gradually remove the glucose residues at positions C-3 and C-20	Bioavailability: Significantly increased Lipophilicity: Increased Neuroprotective activity [89,90]
Ginsenoside Re	Ginsenoside Rh1	Lactobacillus spp; Bacteroides	β-glucosidase; α-rhamnosidase	Ginsenoside Re → Ginsenoside Rg2 → Ginsenoside Rh1 First remove the rhamnose group at the C-20 position, then remove the glucose group	Enhanced anti-inflammatory activity [91]
Ginsenoside Rb2	**C**ompound Y	Prevotella spp; Bacteroides spp	α-arabinosidase; β-glucosidase	Ginsenoside Rb2 → Ginsenoside Compound O → Ginsenoside Compound Y	Gradually shorten the sugar chains to increase lipid solubility Achieve specific immunomodulatory activity [92]
Ginsenoside Rf	Ginsenoside Rh1	Microbacterium sp	β-glucosidase	Ginsenoside Rf → Ginsenoside Rh1	Increased permeability of the blood-brain barrier [93,94]

### 5.2 Novel formulations and delivery systems

(1) The application prospects of nanoscale delivery systems

**Fig. 7:**
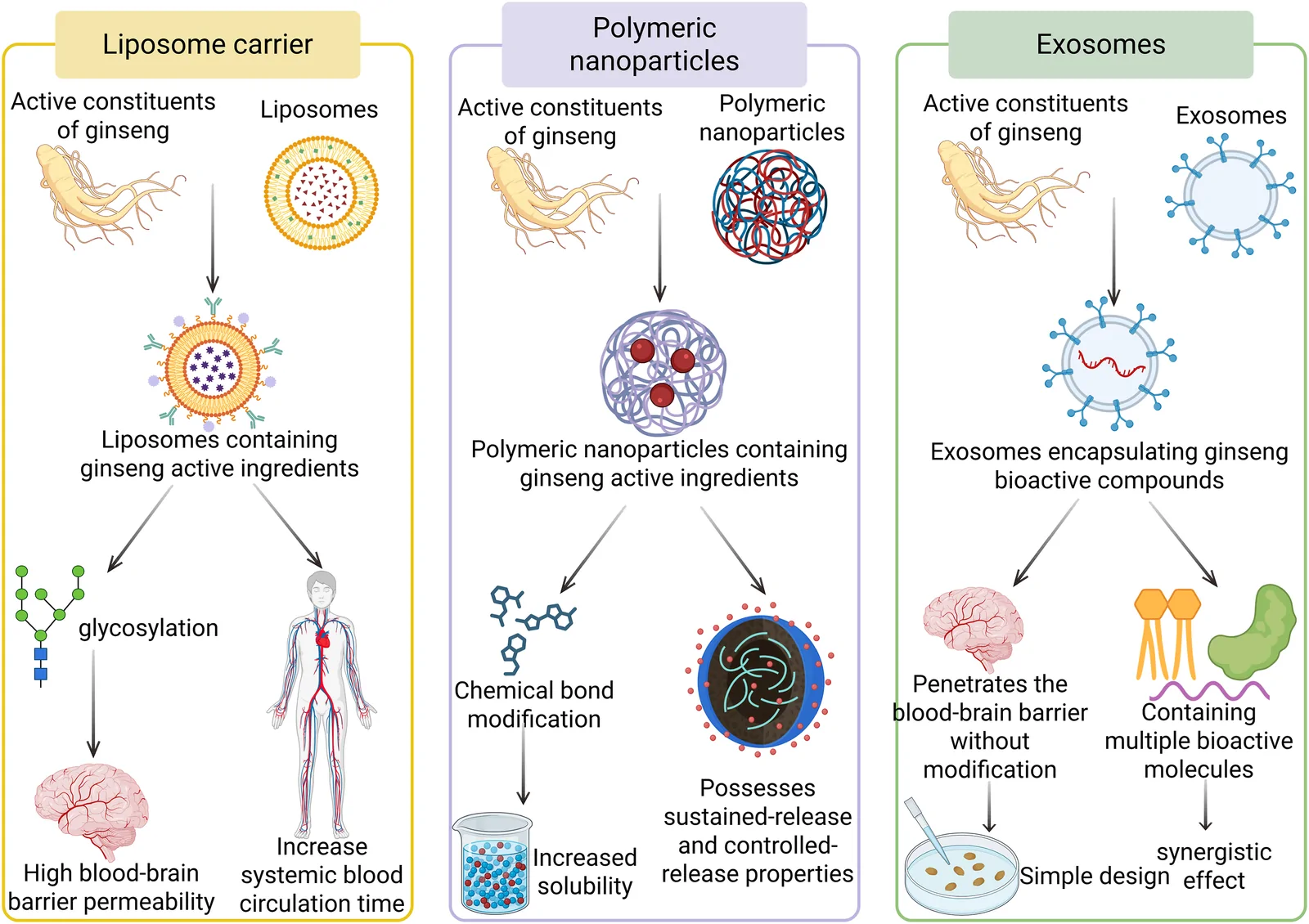
**Strategies for enhancing the bioavailability and penetration of ginseng active components via nano delivery systems.** Utilizing nanocarriers such as liposomes, polymeric nanoparticles, and exosomes can significantly improve the bioavailability and blood-brain barrier penetration of ginseng active components. Liposomes enhance component stability, boost blood-brain barrier penetration efficiency through glycosylation modification, increase brain accumulation, and prolong systemic circulation time. Polymeric nanoparticles chemically modify ginsenosides to improve water solubility while enabling sustained and controlled release. Exosomes inherently possess blood-brain barrier -crossing capability without requiring complex modifications; beyond delivering active components, their inherent lipids, proteins, and miRNAs synergistically enhance therapeutic effects against AD.

In recent years, nanocarrier technology has enhanced the bioavailability and brain targeting of ginseng's active constituents. Liposomal carriers improve ginsenoside stability and lipophilicity, prolong circulation time, facilitate blood-brain barrier crossing, and enhance brain accumulation [98]. Polymeric nanoparticles encapsulate ginsenosides (due to poor water solubility) in a hydrophobic core or via chemical bonding, improving solubility and stability in aqueous environments and preventing premature degradation; the polymer matrix also enables sustained, controlled release, avoiding blood concentration fluctuations [99]. Exosome nanoparticle delivery, an emerging approach, offers excellent biocompatibility and brain-targeting properties. Exosomes can cross the blood-brain barrier without modification and are preferentially taken up by microglia and neurons, providing a natural advantage for modulating neuroinflammation. By shifting microglial polarization from M1 to M2, they enable precise immunomodulation, facilitating recovery in AD. Moreover, ginseng exosome nanoparticles contain not only ginsenosides but also lipids, proteins, and miRNAs, which exert synergistic effects, yielding more comprehensive therapeutic outcomes than single-component approaches (Fig. 7) [100].

(2) Establishing a multifunctional delivery system

Future intelligent delivery systems may employ polymer conjugation or micellar encapsulation to convert hydrophobic ginsenosides into water-dispersible forms, enhancing solubility and bioavailability. Modified carriers (e.g., transferrin receptor antibodies, acetylcholine analogues) can enable blood-brain barrier crossing for neuroprotective therapy [101]. Smart response mechanisms—pH-responsive, temperature-responsive, enzyme-responsive—trigger carrier disintegration under specific conditions, enhancing safety and therapeutic efficacy [102].

## 6 Conclusion

The pathophysiological mechanisms of AD involve multiple pathways, including Aβ deposition, abnormal phosphorylation of tau protein, neuroinflammation, oxidative stress, and a deficiency in neurotrophic factors. Neurotrophic factors play a critical role in maintaining neuronal survival, synaptic plasticity, and cognitive function; however, their expression is reduced and their function is impaired in the brains of patients with AD. The active components of ginseng regulate the neurotrophic factor network through multiple targets and pathways, establishing clear mechanistic links with the core pathological features of AD: Ginsenoside Rg1 primarily upregulates BDNF, activating the TrkB/PI3K/Akt signaling pathway. This not only improves synaptic plasticity but also inhibits Aβ-induced neuronal apoptosis and reduces the excessive phosphorylation of tau protein at Ser396/404, thereby counteracting Aβ toxicity and tau pathology. Ginsenoside Rb1 enhances NGF signaling, promotes the survival and axonal growth of basal forebrain cholinergic neurons, counteracts Aβ-mediated downregulation of the NGF receptor TrkA, and indirectly reduces tau phosphorylation by modulating GSK-3β activity, thereby maintaining cytoskeletal integrity. Ginsenosides Rg3, Rc, Rd, and other ginsenoside components possess anti-inflammatory and antioxidant effects. By inhibiting the NF-κB pathway and reducing ROS levels, they protect neurotrophic factors from oxidative degradation while promoting glial cell-derived GDNF expression and improving the neuronal microenvironment. Ginseng polysaccharides and non-saponin components synergistically increase the expression of NT-3 and GDNF: By modulating the polarization state of microglia, they alleviate the inhibitory effects of chronic neuroinflammation on the neurotrophic factor system, thereby indirectly maintaining the stability and activity of BDNF and NGF.

However, the clinical translation of ginseng still faces significant challenges: 1. Poor penetration of the blood-brain barrier: Most ginsenosides have limited distribution in the brain, making it difficult to achieve effective concentration in the brain following oral administration. 2. Low bioavailability: Ginsenosides are metabolized in the gut and are unstable; they undergo significant first-pass metabolism, and variations in gut microbiota among individuals lead to inconsistent conversion efficiency. 3. Formulation and dosage limitations: Most existing studies use single-component or non-standardized extracts, and lack data on ingredient ratios, dose-response relationships, and in vivo pharmacokinetic parameters that meet drug development standards, making it difficult to replicate and compare research results. 4. Insufficient clinical evidence: Most current studies are limited to cellular and animal models; high-quality randomized controlled trials in humans are rare, and there is a lack of clinical endpoint evaluations using neurotrophic factors as biomarkers.

Future research should focus on the following areas to deepen our understanding of the mechanisms by which ginseng-modulated neurotrophic factors intervene in AD and to facilitate clinical translation:1. The systematic integration of multi-omics technologies: A single omics approach is insufficient to fully elucidate the complex, multi-component, and multi-target regulatory network of ginseng. Future research should integrate transcriptomics, proteomics, network pharmacology, and metabolomics to systematically reveal the regulatory mechanisms by which the bioactive components of ginseng modulate the neurotrophic factor network. Using transcriptomic RNA-seq analysis, we will investigate the differential expression of BDNF, NGF, GDNF, and their receptors in brain tissue from AD models before and after ginseng treatment and identify key transcription factors within co-expression networks. Through proteomics, we will identify key phosphorylation sites in ginsenoside-regulated signaling pathways to clarify their direct targets. Using network pharmacology, construct a multi-level network linking “ginseng bioactive components—potential targets—AD-related pathways—neurotrophic factors” to predict key functional nodes. Combine this with molecular docking and molecular dynamics simulations to screen for high-affinity targets. Analyze changes in brain and peripheral metabolite profiles following ginseng treatment via metabolomics to elucidate the relationship between the restoration of neurotrophic factor function and metabolic remodeling. 2. Mechanisms of the ginseng-nerve growth factor interaction mediated by the gut-brain axis: The gut microbiota plays a critical role in the biotransformation of ginsenosides, and the gut-brain axis serves as a vital bridge linking the regulation of neurotrophic factors between the peripheral and central nervous systems. Future research should focus on exploring how the gut microbiota converts natural ginsenosides into more bioactive rare ginsenosides, and on comparing the effects of differences in conversion efficiency among individuals with varying microbial compositions on the expression of neurotrophic factors. Furthermore, it is necessary to elucidate how the active components of ginseng, following metabolism by the gut microbiota, influence BDNF/TrkB signaling in central nervous system cells via the intestinal epithelial barrier, the vagus nerve, or the circulatory system, and to utilize non-targeted metabolomics to identify key metabolites produced by the microbiota in plasma and cerebrospinal fluid following ginseng intervention. Through these studies, ginseng promises to evolve from a traditional medicine into a new, evidence-based, multi-target intervention strategy for AD.

## Author contributions

Qucheng Huang: Writing – review & editing, Writing – original draft, Software, Investigation. Yining Xie: Software, Data curation. Xiaojun Zhang and YuanYuan Zuo: Software, Investigation. Leiyi Wang: Software, Investigation. Hewei Xu: Software. Miao Yu: Investigation. Chang Liu: Writing – review & editing.

## Conflicts of interest statement

The authors declare no conflict of interest.
